# An application of computable biomedical knowledge to transform patient centered scheduling

**DOI:** 10.1002/lrh2.10393

**Published:** 2023-09-19

**Authors:** Namita Azad, Carolyn Armstrong, Corinne Depue, Timothy J. Crimmins, Jonathan C. Touson

**Affiliations:** ^1^ Columbia University Irving Medical Center New York New York USA

**Keywords:** algorithm, innovation, patient‐centered, scheduling

## Abstract

**Introduction:**

Efficient appointment scheduling in the outpatient setting is challenged by two main factors: variability and uncertainty leading to undesirable wait times for patients or physician overtime, and events such as no‐shows, cancellations, or walk‐ins can result in physician idle time and under‐utilization of resources. Some methods have been developed to optimize scheduling and minimize wait and idle times in the inpatient setting but are limited in the outpatient setting.

**Methods:**

People and Organization Development, an internal group of organizational developers, led the development of a solution that selects the optimal group of appointments for a patient that minimizes the time between associated procedures as well as lead time built using a linear integer program. This program takes appointment requests, availability of resources, order constraints, and time preferences as inputs, and provides a list of the most optimal groupings as an output. Included in the methodology is the technical infrastructure necessary to deploy this within an electronic medical record system.

**Implementation and Test Plan:**

A pilot has been designed to run this algorithm in a single department. The pilot will include training staff on the new workflow, and conducting informal interviews to gather qualitative data on performance. Key performance indicators such as schedule utilization, resource idle time, patient satisfaction, average appointment lead time, and average waiting time will be closely monitored.

**Discussion:**

The model is limited in accounting for variability in appointment length potentially resulting in inaccurate schedules for healthcare providers and patients. Future states would incorporate certain visit types starting through machine learning techniques. Additionally expanding our data pipeline and processing, developing greater communication software, and expanding our research to include other departments and subspecialties, will enhance the accuracy and flexibility of the algorithm and enable healthcare providers to provide better care to their patients.

## 1 INTRODUCTION

Efficient appointment scheduling in the outpatient setting is challenged by the inherent variability and uncertainty associated with patient flow. Variability in appointment length and patient arrivals, including events such as no‐shows and cancellations, compromise the scheduling process and can lead to undesirable wait times for patients. This variability can also result in physician idle or over‐time, and under‐utilization of resources. In this context, a “resource” refers to any entity within the healthcare system that has its own schedule and/or inventory, and is available in the scheduling system. Examples of resources include providers, diagnostic tests, imaging machines, exam rooms, or any other element that is necessary for a specific booking, which may require more than one resource. To maximize efficiency and patient and provider and experience in the healthcare setting, scheduling solutions ought to assign resources while accounting for the variability that encompasses both the uncertainty in service duration, as well as in the number of patients that will be served within a given time period.^1^

Within scheduling workflows, there is also the challenge of handling administrative rules such as ensuring patients are booked with providers that par with insurance which can vary even for a single provider depending on which location a patient is seeing them at making it an essential consideration for an advanced scheduling system. Additionally, incorporating patient preferences such as location ought to become more streamlined however, many of these preferences are still being discussed at the point of scheduling. Utilizing historic patient preference data and accounting for factors such as location by calculating distance from residence to possible sites would benefit both scheduling staff and patient experience. Existing studies have primarily focused on scheduling optimization in the inpatient,^2^ however, several articles have explored the challenges and opportunities in appointment scheduling within the outpatient setting, shedding light on potential solutions and approaches.

Recent studies have shown the significance of incorporating patient preferences and provider availability in designing appointment scheduling systems for ambulatory care services^3^ and the need to focus on the overall design and considerations for scheduling systems, emphasizing the need to consider patient flow dynamics, no‐show rates, and service time variability as well as patient preferences. However, the specific scheduling of multiple appointments at once and the proximity of those appointments in time are not explicitly addressed.

An optimal outpatient appointment scheduling approach that addresses factors such as varying appointment lengths, patient preferences, and stochastic patient arrivals^4^ has been addressed to develop an optimization model, there continues to be a gap in addressing patients scheduling multiple appointments at once or attempt to schedule those appointments close in time. Dynamic appointment scheduling of a stochastic server with uncertain demand in a 2016 study,^5^ provided further understanding of the important trade‐offs in schedule efficiency and access to service when implementing overbooking,^6^ but each lacks multiple appointment booking features as well.

A coordinated patient appointment scheduling for a multi‐station healthcare network^7^ can offer the opportunity to optimize scheduling decisions across multiple locations, considering factors such as patient preferences, time‐dependent service durations, and resource constraints. This work is important for consideration of more complex service solutions as it emphasizes coordination of appointments at multiple sites, but it does not explicitly investigate patients scheduling multiple appointments at once or the proximity of those appointments.

Related to our goal of capacity balancing across resources, Berg et al. present a method for balancing provider schedules in outpatient specialty clinics.^8^ The study addresses the challenge of workload imbalance among providers and proposes a method to balance schedules by redistributing patients among providers. However, it also leaves out patients who require complex care or those seeking to book several proximate appointments at once. In contrast, the literature review by Marynissen and Demeulemeester, “Literature review on multi‐appointment scheduling problems in hospitals,” provides a comprehensive overview of the various multi‐appointment scheduling problems in the hospital context, which offers valuable insights for addressing the challenges of scheduling multiple appointments in the outpatient setting.^9^ Lastly, the study by Robinson and Chen, “A comparison of traditional and open‐access policies for appointment scheduling” provides insights into how open‐access policies can be key for designing effective appointment scheduling systems in the outpatient setting.^10^

Incorporating insights from research done in this space has supported the development of an outpatient scheduling optimization algorithm that would provide valuable guidance for addressing the challenges of variability, uncertainty, patient preferences, and resource balancing in appointment scheduling. Within the growing body of outpatient optimization work, a program is yet to be developed in the ambulatory setting that has the ability to serve patients requiring multiple appointments, potentially spanning multiple specialties and resources, while also incorporating robust patient preferences and administrative rules. Furthermore, certain attempts to optimize scheduling using patient data have been proven to exacerbate racial health disparity by assuming no show rates used in their model are independent of patient characteristics.^11^

Through collaboration with frontline staff, Columbia University Irving Medical Center's (CUIMC) People and Organization Development (POD), an internal group of organizational developers, identified the unmet need for a patient centered and optimized scheduling solution. The POD team utilized a novel framework, the Innovator Accelerator Methodology (IAM), to assess the viability and organize the development of a solution. The first steps included creating an assessment of the care gap through a combination of on‐site observation with schedulers, informal interviews with department administrators and practice managers and meetings with senior medical officers, as well as leaders in Operations and Information Technology at Columbia. After outlining current state workflows and performance, an idea assessment was brought to our Clinical Innovation Advisory Group (CIAG), a panel of clinicians with diverse expertise that provides guidance to our Clinical Innovation Lab (CIL) administrators on the impact of proposed innovations. The CIAG panel evaluates proposed innovations using a scoring system that rates the potential benefits based on three basic areas: the level of burden the innovation would eliminate, the total cost of resources required, and the panel's general level of excitement around the idea. The scoring scale ranges from one to five, with proposals that receive higher scores being considered for further evaluation. Once proposals have been selected to advance past the CIAG scoring process, the CIL helps to connect projects to specialists throughout the organization who may be able to support the project. In the case of the scheduling optimization initiative, the CIL connected a robust group of technical specialists from the Department of Biomedical Engineering, leaders in Operations, and some expert schedulers and providers who were willing to help develop and/or advise on practical solutions.

Through the IAM, to fill the gap in outpatient scheduling without amplifying inequity, the POD team designed an algorithm that selects the optimal group of appointments for a patient which minimizes the time between associated procedures as well as lead time. Additionally, we maintain that the program be flexible to patient and physician preferences, which many optimization approaches neglect, though these factors appear frequently in realistic problems and implicate the applicability of any model. Within the Learning Health System's scope of Computable Biomedical Knowledge (CBK) this solution falls into the category of algorithms that transform data in a deliberative process, making previously human‐reliant processes and decisions automated and optimized for a quicker and more effective scheduling performance.

## 2 METHODS

With the intention of creating a global solution that respects local wisdom, our development process began with rigorous interviews with scheduling and operations staff. These are people who either directly schedule patients or are familiar with how schedules are uploaded, maintained, and accessed within the Electronic Health Record (EHR). These interviews informed and confirmed what our hard and soft constraints would be, which relate to characteristics such as job length, spread and sequence of visits, and whether these visits all get scheduled in 1 day. This interview process was also imperative for the discovery of what technical infrastructure would be necessary to 1. feed the necessary data to our model and 2. allow our model to interact in real time with the EHR and schedule these appointments.

Given the constraints of scheduling in the EHR, some of which are technical while others more tenacious (e.g., clearance within the EHR to schedule across departments), it was important to us that our model would be agnostic to both the user and to the resources being scheduled. This is made possible through APIs to scrape resource availability data from the EHR and then store and process it externally. Once the solution, or optimal grouping of appointments, is generated, our software books these appointments within the EHR.

In programming the interface, it was important to include areas for inputs such as date and time preference, visit sequence rules, and location preference, as these may vary on a patient or visit type basis, and with over 230 specialties at some institutions, and any combination of visit types possible, trying to configure and maintain some of these rules in advance is an arduous and insufficient way to manage group appointment scheduling. Once all this technical infrastructure was in place, we were able to integrate the optimization model that would provide the output of optimal appointment groupings.

Our optimization model makes use of a popular open‐source linear programming library in Python called PuLP. This library has applications across resource allocation, transportation, blending, scheduling, and many other domains and enables us to easily define our problem in terms of decision variables, objective function, and constraints. Because it may not always be feasible or most favorable for the patient, the model utilizes one of two functions based on the scheduler's input: one for single‐day scheduling and another for multi‐day scheduling.

The first function's objective function is a combination of minimizing the spread time, minimizing the total travel time, and minimizing the difference between appointments scheduled and appointments preferred by the patient—single‐day schedule options are returned. The second function's objective function is a combination of minimizing the number of scheduled days, minimizing lead time, and maximizing the available capacity—multi‐day schedule options are returned. For our study, we use these either of these functions depending on a specific patient's need and preference, though it is possible to augment the scheduling program so that for a patient who is willing and able to be seen on one or multiple days, a scheduler could use these functions in a multi‐objective fashion with a certain weight given to each objective based on your institution's ideal.

The PuLP model also incorporates constraints to ensure that appointments do not exceed the available capacity of selected resources, and that the start time of each appointment does not violate any previous appointment's end time. Additionally, constraints ensure that total travel time between resources does not exceed the resource's time available, and that appointments are scheduled during a patient or scheduler's preferred time slot if provided. To demo this solution yourself, please visit https://github.com/PODcarmstrong/schedulingoptimization. This repository contains an annotated Jupyter notebook and a text file describing how to use it. At the time of this paper's submission, the demo was last run on Python 3.10.12 and the package versions NumPy 1.23.5, PuLP 2.7.0, and Matplotlib 3.7.1. Please see Table 1a and 1b below for more detailed equations describing the model.

**TABLE 1a:** Single‐day solution.

Decision variables	$X_{\text{rsrc}}=\left\{0,1\right\},\text{whether to schedule resource rsrc to appointment window}\;w$
Objective function	$\left(a*\text{spread time}+b*\text{travel time}+c*\mathrm{abs}\_\text{preferred}\_\mathrm{ts}\_\text{diff}\right)$ *Minimize spread time*: spread_t=end_t−start_t $\text{start}\_t\leq X_{\text{rsrc},w}*w*T\_{\mathrm{VT}}_{\mathrm{vt}}+\mathrm{ST}\_\mathrm{DE}P_{\mathrm{dep}}+\left(1-\sum_{w=0}^{N\_W_{\mathrm{vt}}}X_{\text{rsrc},w}\right)*M,\text{rsrc}=1,\ldots ,N\_\text{RSRC},w=1,\ldots ,N\_W_{\mathrm{vt}}$ $\mathrm{end}\_t\geq X_{\text{rsrc},w}*w*T\_{\mathrm{VT}}_{\mathrm{vt}}+X_{\text{rsrc},w}*\mathrm{JOB}\_\mathrm{LE}N_{\mathrm{vt}},i=1,\ldots ,N\_\text{RSRC},j=1,\ldots ,N\_{\mathrm{TS}}_{\mathrm{vt}}$ *Minimize travel time*: $\text{total}\_\text{travel}\_t=\sum \text{travel}\_t_{{\text{rsrc}}_{i},{\text{rsrc}}_{j}}$ $\text{travel}\_t_{{\text{rsrc}}_{i},{\text{rsrc}}_{j}}\geq Y_{vt_{i},vt_{j}}*\mathrm{DI}S_{{\mathrm{dep}}_{i},{\mathrm{dep}}_{j}}-\left(1-X2_{{\text{rsrc}}_{i}}\right)*M-\left(1-X2_{\mathrm{rsr}c_{j}}\right)*M$ $Y_{{\mathrm{vt}}_{i},{\mathrm{vt}}_{j}}=\left\{0,1\right\},\text{whether to schedule visit type}\;{\mathrm{vt}}_{i}\;\text{just before visit type}\;{\mathrm{vt}}_{j}$ $\sum_{{\mathrm{vt}}_{i}=0}^{N\_\mathrm{VT}}Y_{{\mathrm{vt}}_{i},{\mathrm{vt}}_{j}}=1,\sum_{{\mathrm{vt}}_{j}=0}^{N\_\mathrm{VT}}Y_{{\mathrm{vt}}_{i},{\mathrm{vt}}_{j}}=1,Y_{{\mathrm{vt}}_{i},{\mathrm{vt}}_{i}}=0$ $Y_{{\mathrm{vt}}_{i},{\mathrm{vt}}_{j}}+Y_{{\mathrm{vt}}_{j},{\mathrm{vt}}_{i}}<=1,\sum \sum Y=N\_\mathrm{VT}-1$ $X2_{\text{rsrc}}=\left\{0,1\right\},\text{whether resource rsrc is scheduled}$ $X2_{\text{rsrc}}=\sum_{w=0}^{N\_W_{\mathrm{vt}}}X_{\text{rsrc},w}$ *Minimize the difference between scheduled time slots and preferred time slots*: $\text{pref}\_\text{diff}=\sum |\sum_{w=0}^{N\_W_{\mathrm{vt}}}X_{\text{rsrc},w}*w*T\_{\mathrm{VT}}_{\mathrm{vt}}-\mathrm{ts}\_\text{pref}|$ *Maximize the capacity of the days being scheduled*
Constraints	*Resource Constraints*—appointments should be scheduled for the day available $X_{\text{rsrc},w}\leq \mathrm{Ca}p_{\text{rsrc},w}$ *Demand Constraints*—total number of appointments should be the same as requested ∑resourceifor visit typevtX2rsrc=Reqvt,vt=1,…,N_VT *Time Constraints*—the t + 1_th appointment should be scheduled later than the t_th appointment (considering job length, travel time, and interval constraints) $\mathrm{ST}\_\mathrm{DE}P_{\mathrm{de}p_{i}}+\sum_{w_{i}=0}^{N\_W_{vt_{i}}}X_{{\text{rsrc}}_{i},w_{i}}*w_{i}*T\_{\mathrm{VT}}_{vt_{i}}+\mathrm{JOB}\_\mathrm{LE}N_{vt_{i}}+\mathrm{DI}S_{\mathrm{de}p_{i},\mathrm{de}p_{j}}+\mathrm{Int}v_{vt_{i},vt_{j}}\leq$ $\mathrm{ST}\_\mathrm{DE}P_{\mathrm{de}p_{j}}+\sum_{w_{j}=0}^{N\_W_{vt_{j}}}X_{{\text{rsrc}}_{j},w_{j}}*w_{j}*T\_{\mathrm{VT}}_{vt_{j}}+\left(1-Y_{vt_{i},vt_{j}}\right)*M+\left(1-X2_{{\text{rsrc}}_{i}}\right)*M+\left(1-X2_{{\text{rsrc}}_{j}}\right)*M$ *Order constraints*— $Y_{{\mathrm{vt}}_{i},{\mathrm{vt}}_{j}}<=\text{order}\_\mathrm{pre}f_{vt_{i},vt_{j}}\;\text{if order}\_\mathrm{pre}f_{vt_{i},vt_{j}}!=-1$

**TABLE 1b:** Multi‐day solution.

Decision variables	$X_{\mathrm{vt},d}=\left\{0,1\right\},\text{whether to schedule visit type}\;\mathrm{vt}\;\text{to}\;\mathrm{day}\;d$ *For order preference*: $Y_{{\mathrm{vt}}_{1},{\mathrm{vt}}_{2}}=\left\{0,1\right\},\text{whether to schedule visit type}\;{\mathrm{vt}}_{1}\;\text{after visit type}\;{\mathrm{vt}}_{2}$
Objective function	*Minimize the number of scheduled days*: $D1_{d}=\sum_{\mathrm{vt}=0}^{N\_\mathrm{VT}}X_{\mathrm{vt},d},\#\text{of appointments scheduled to}\;\mathrm{day}\;d$ $D2_{d}=\left\{0,1\right\},\text{whether there}\;\mathrm{are}\;\text{appointments scheduled to}\;\mathrm{day}\;d$ $N\_\text{scheduled}\_d=\sum_{d}^{N\_D}D2_{d}$ *Minimize leading time*: *using average leading time here (sum of leading time/# of appointments)* $D_{\mathrm{vt}}=\sum_{d=0}^{N\_\mathrm{DAY}}X_{\mathrm{vt},d}*d$, visit type vt is scheduled to day d *Maximize the capacity of the days being scheduled*
Constraints	*Resource Constraints*—appointments should be scheduled for the day available $X_{\mathrm{vt},d}\leq \mathrm{Ca}p_{\mathrm{vt},d}$ *Demand Constraints*—total number of appointments should be the same as requested ∑Xvt,d<=Reqvt *Scheduled Days Constraints*—Define D2, if $D2_{d}=0$, $D1_{d}=0$; else no restriction for $D1_{d}$ $D1_{d}<=D2_{d}+D2_{d}*M$ $D1_{d}<=D2_{d}-D2_{d}*M$ *Order constraint*— $D_{vt_{i}}<=D_{vt_{j}}+\text{order}\_{\text{pref}}_{{\mathrm{vt}}_{i},{\mathrm{vt}}_{j}}*M$ $D_{vt_{i}}>=D_{vt_{j}}-\left(1-\text{order}\_{\text{pref}}_{{\mathrm{vt}}_{i},{\mathrm{vt}}_{j}}\right)*M$ *Same‐Day Constraint (some of the appointments must be scheduled on the same day)* ‐ $D_{vt_{i}}=D_{vt_{j}}$

**FIGURE 1:**
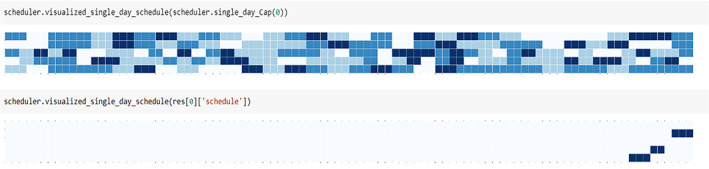
Single‐day schedule visualized solution.

Note that capacity values for each department are randomly generated in the first draft of the model. Capacity values are generated for each visit type and resource in each window. Using simulated resource availability and patient request data, we have successfully visualized optimal schedule solutions using the libraries Matplotlib and Seaborn (see Figure 1 below). In implementation, schedulers will have the option to visualize multiple options for any given patient request so that when the first optimal option may not be feasible or favorable to the patient, we can choose an equally or almost optimal option during different windows.

## 3 IMPLEMENTATION AND TEST PLAN

To test the efficacy of the scheduling optimization algorithm, a pilot has been designed for implementation in a single department, the Department of Cardiology, which has several subspecialties and a patient population often requiring various procedural tests and lab services. The pilot will involve deploying the algorithm for a period of 3 months and monitoring several key metrics to assess its impact.

An important part of deployment will be the training of staff on the new workflow, which will be facilitated by the POD team. Developers, department leadership, and POD leadership will govern the pilot collaboratively, while developers will handle technical maintenance and POD will support staff as they acclimate to the new workflow. Informal interviews will be conducted throughout the pilot to gather qualitative data on performance. During the pilot, we will also closely monitor key performance indicators including schedule utilization, resource idle time, patient satisfaction, average appointment lead time, and average waiting time. These metrics will be used to evaluate the effectiveness of the scheduling optimization algorithm and to identify areas for improvement.

Following the pilot, we will conduct a thorough performance review to assess the algorithm's impact on the department's overall operations. This review will include an evaluation of the key metrics mentioned earlier and an assessment of the algorithm's scalability, cost‐effectiveness, and ease of use. Based on the results of this review, we will determine whether to proceed with a full‐scale implementation of the scheduling optimization algorithm across the entire department.

## 4 DISCUSSION

Our scheduling optimization algorithm provides a valuable tool for healthcare providers to address the challenges of scheduling appointments in a busy clinical environment. However, like any computational model, our algorithm has its limitations. One of the most significant limitations is the fact that the model does not yet account for the variability in appointment length. As a result, the algorithm may not always produce the most accurate schedule for healthcare providers and patients.

To address this limitation, we plan to investigate how to incorporate the likelihood of certain visit types starting or ending late through machine learning techniques. For instance, we could examine the historical data of each appointment type to determine the probability of a particular appointment starting or ending late, and then integrate this information into the algorithm. This approach could help healthcare providers create more accurate schedules that better reflect the reality of their clinical environment.

Another way to improve our scheduling optimization algorithm is to expand our data pipeline and processing to include more location‐level features or examine the role of time of day or day of the week in an appointment or travel time variability. By considering these factors, we can create a more sophisticated algorithm that considers the unique needs and characteristics of each clinical environment. This approach could help healthcare providers develop more efficient schedules that improve patient outcomes and satisfaction, especially for distinct populations, such as patients who are wheelchair‐bound. By utilizing patient characteristics and location accessibility route features, we can improve equity within optimized schedules.

In addition, we believe that scheduling optimization could be expanded through greater communication software, which could enable dynamic schedule adjustments when unexpected events occur. For instance, patients could be given the option to come early or late when necessary, or they could be provided alternative travel routes or notified when severe weather events happen. Such software would allow patients and healthcare providers to respond to unexpected events in a more efficient and effective manner.

Finally, we recognize that our current model is only designed to optimize schedules for a specific department with a limited number of subspecialties. Going forward, we plan to expand our research to include other departments and subspecialties. By doing so, we can develop a more comprehensive understanding of scheduling challenges across different clinical environments and identify new opportunities for optimization.

In conclusion, while our scheduling optimization algorithm provides an effective means of addressing scheduling uncertainty, there is still significant room for improvement. By incorporating machine learning techniques, expanding our data pipeline and processing, developing greater communication software, and expanding our research to include other departments and subspecialties, we can enhance the accuracy and flexibility of the algorithm and enable healthcare providers to provide better care to their patients.

## CONFLICT OF INTEREST STATEMENT

The authors declare that they do not have any conflicting interests.

## Supporting information


**CBK Artifact S1:** LHS optimized scheduling demo.
